# Women's Perspectives on Men's Role in Addressing Violence Against Women: Insights from Lived Experience and Practice

**DOI:** 10.1177/10778012251362237

**Published:** 2025-07-30

**Authors:** Jessica Wild

**Affiliations:** 1 School of Social Sciences, 4921 University of Westminster, London, UK

**Keywords:** VAW prevention, engaging men, lived experience, discourse analysis

## Abstract

This paper explores men's participation in efforts to address men's violence against women and minoritized genders, through the dual lens of women who have lived experience of domestic violence and/or abuse and that of practitioners working in domestic violence and/or abuse support or refuge services in England. Data substantiate the imperative of men's engagement in antiviolence against women and minoritized genders practice and activism while also surfacing the epistemological, practical, and political complexities it entails, especially when framed by “men listen to men” discourses. The paper argues that men's participation can disrupt victim-blaming discourses and promote men's increased responsibilization to achieve structural change. Crucially, the paper underscores the importance of consistently centering the lived experience epistemologies of victim–survivors.

## Introduction

In 2020 and 2021, a series of widely documented femicides in England forced the public and academic debates regarding men's role(s) in ending violence against women and minoritized genders (VAW) back into the public consciousness as many women asked, “What are men doing about men's continued violence against women?” The murders of Nicola Smallman, Bibaa Henry, Sarah Everard, and Sabina Nessa represent only a fraction of the women killed every year in England by men, and there have been several more during and since this paper has been written. The deaths of the women named here received particularly intensive, high-profile media coverage, sparking public outcry and widespread national protest across England (Topping, 2022) to a degree arguably not seen since the 2017/2018 ascendance of Tarana Burke's “me too” campaign in the United States, United Kingdom, and elsewhere. The public response to these women's murders served as a stark reminder of the ubiquity of men's VAW and of the urgent need to continue to develop more radical and transformative approaches that shift responsibility for ending men's VAW away from women and survivors and onto men, those that harm, as well as society more broadly.

The onus for addressing men's violence, especially sexual and domestic violence and/or abuse (S/DVA), has typically fallen upon women and those with lived experience of gender-based violence (GBV). Their responsibilization occurs and is sustained at various scales—through leading social action campaigns, delivering services for those who have experienced abuse, or individually managing the abusive behaviors of intimate partners, especially when their families come to the attention of children's social care, as data from this study have shown (Wild, 2022). Moreover, women are frequently “advised” to take measures to protect themselves against potentially predatory or violent men, known or unknown, thereby further embedding the notion that women must take charge of the problem of men's violence(s). Advisory practices aimed at women and girls erroneously imply that they have a degree of agency concerning perpetrators’ actions and, in turn, contribute to a well-established victim-blaming narrative, which has long underpinned the dominant discourse patterning VAW (Dunn & Powell-Williams, 2007). This discourse can embolden men who perpetrate violence, and it mirrors the responsibilizing tactics they routinely deploy (Pain, 2021). At its core is the construction of gendered violence as exclusively a “women's issue” (Crooks et al., 2007), thereby curtailing the extent to which men perceive it as a matter connected to themselves, gender inequalities, and/or the corresponding performance of certain gender (hetero)norms (Boyle, 2019; Casey et al., 2017).

The role(s) men (can, do or should) occupy in preventing VAW and GBV more broadly has been a field of enquiry for at least the last four decades (Messner et al., 2015; Storer et al., 2016). Central to men's participation in anti-VAW prevention is the (re)conceptualization of men not only as possible perpetrators of gendered violence but also as potential partners, “allies” or collaborators in efforts to eliminate men's violence(s) and to promote gender equality (Casey, 2010; Collins, 2000; Flood, 2015; Tolman et al., 2016; Westmarland et al., 2021). This has led to debates regarding how men's involvement is best operationalized to maximize potential gains and minimize potential harms (Peretz, 2020). Droogendyk et al. (2016) note that “helping” across boundary lines of difference, such as in this case, is not always a “benign” activity. In the context of efforts to address men's VAW, helping is inextricably bound up with hierarchies of epistemological privilege and intersectional gender politics (Casey et al., 2013). This linkage is especially pronounced when it comes to the construction of men as “allies” (Casey, 2010), in part because “allyship” does not occur outside of extant intersectional gender hierarchies, which remain present even in anti-VAW movement and practice spaces, as interviews with men as part of this study illustrate (Wild, 2023). Against this backdrop, this paper focuses on the participation of men as agents for change in practice and activism to reduce or intervene in VAW. The men who took part in this study occupied a range of roles, including activist and campaigning roles, leadership and commissioning roles responsible for VAWG strategy, social workers, and domestic abuse practitioner roles (for more detail see, Wild, 2023). For ease, they are referred to here as “engaged men” (Burrell & Flood, 2019; Tolman et al., 2016). The need for and relevance of men's involvement in this space is critically analyzed through the dual lens of women practitioners operating in the field of DVA prevention and intervention in England and that of women who have lived experience of men's violence.

There is a notable paucity of research examining how these two groups conceptualize “engaged men” and their role(s) in challenging men's VAW, as well as how they experience the diversification of the movement and sector to include (more) men. This paper, therefore, contributes to a comparatively small but growing body of empirical research on the topic (see for example, Castellino, 2014; Macomber, 2015; Peretz, 2018a; Wiley & Dunne, 2019). Discourse analysis indicates how participant accounts tap into some of the more intractable questions concerning the role of men as antiviolence agents for change—first, *who* is best placed to facilitate men and boys’ engagement? Second, is it necessary (or indeed appropriate) to instrumentalize men's epistemic and social privilege to expand the possibilities for men's participation in antiviolence efforts based on a discourse that men listen to other men more readily? In considering these questions, it is not the intention of this paper to argue against men supporting the efforts of women and minoritized genders in eradicating gendered violence. Instead, the arguments offered here move beyond an interrogation of *if* men have a part to play in eliminating men's violence, to *what* that role looks like and how it is most productively and respectfully enacted to reduce men's VAW and improve outcomes for victim–survivors. The paper argues for an approach to men's antiviolence participation that is accountable and attuned to the complex workings of unequal gender and power dynamics. It must also foreground the lived experience epistemologies of victim–survivors, women and minoritized genders, especially when providing direct intervention to people who have experienced men's violence(s).

The remainder of this paper is structured as follows: an outline of key literature is provided before moving on to the methods used to produce novel data. Following this, the findings are set out and discussed. Analysis conveys the complexities and nuances associated with men's involvement in this space. It also points to the value of an intersectional, coalitional politics to address the extant challenges and barriers to collaboration communicated by the women in the sample and to expand the scope of men's responsibilization in men's violence prevention. With this, is a rejection of excessively narrow notions of “allyship” that can limit the possibilities for social change and gender equality and that do not account for the interlocking structural conditions that foster the perpetration of VAW and GBV more broadly. The paper concludes by asserting that notwithstanding these challenges, men's meaningful involvement in efforts to curtail men's violence is a vital component in the urgent and ongoing work to reduce prevalence rates, transform a culture that continues to permit femicide and, to disrupt a sociocultural discourse that often holds women, minoritized genders, and victim–survivors responsible for men's violence toward them. Attention now turns to a review of the literature concerning “engaged men” in initiatives to address VAW and/or to achieve gender equality, with particular attention to how it has been theorized, understood, and critiqued within feminist scholarship.

## Men in the Movement to End Men's Violence(s)

Men's support of women's efforts to tackle VAW has a reasonably long history, dating back to men's support of the early women's suffrage movement of the mid to late 19th century in Britain (John & Eustance, 1997) and the United States as part of the broader abolitionist movement (Byrd & Guy-Sheftall, 2001). In the 1970s and 1980s, men began proactively responding to women's campaigning against men's violence by advocating for their work in the context of “pro-feminist” activism (Messner et al., 2015). This laid the groundwork for future mobilizations, including in the context of sexual and reproductive health advocacy and antisexism activism. In early 2004, the United Nations Commission for the Status of Women passed a resolution setting out the role of men and boys in achieving gender equality, including the prevention and elimination of GBV (UN, 2004).

The paradigms governing violence against women and girls (VAWG) policy and practice in England have also gradually shifted over time to incorporate men (including as victim–survivors) as the former Conservative government (2010–2024) embedded gender neutrality into its strategic approach to ending VAWG. Evidenced first in the adoption of a gender-neutral definition of DVA in 2012 (Home Office, 2013), this positioning was also reflected in the Domestic Abuse Act (2021). The subsequent Government VAWG strategy (2016–2020) explicitly endorsed the engagement of “men, boys, and bystanders” as “change agents,” “integral” to the prevention of VAWG and the “promotion of social change” (Home Office, 2016, p. 17). But the subsequent strategy (HM Government, 2021) departed from this framing and did not discuss the role(s) of men and boys in VAWG prevention. The Labour Government appointed in 2024 pledged to halve VAWG in the next decade via a “data-driven” strategy targeting known DVA perpetrators (Home Office, 2024).

### Men as “Allies”

One of the most well-embedded concepts used to understand men's participation in the anti-VAW movement is “allyship,” which emerged during the 1990s. It incorporates the key logic that the reduction and prevention of VAW necessitate men's involvement as the group most likely to perpetrate it (Casey & Ohler, 2012; Chabot et al., 2016; Edwards, 2015; Flood, 2011; Schwartz & Dekeseredy, 2008). This produced an important reframing of men as “part of the solution” (Messner et al., 2015, p. 15). “Allyship” in social justice movements (Droogendyk et al., 2016; Kraemer, 2007) is predicated on the notion that institutionalized oppression will persist until those with social advantage work to end it (Casey, 2010). An 'ally' is typically defined as someone from a dominant group who seeks to diminish the systems that grant them privilege (Broido, 2000). In practice, allyship involves a negotiation of different social privileges (Case, 2012; McCorkel et al., 2003), where unearned advantage shapes alliances between groups with diverse social hierarchies. This is evidenced in studies on anti-VAW activism (Case, 2012; Macomber, 2015), antiracist mobilization (Smith & Redington, 2010) and gay liberation movements (Grzanka et al., 2015; Russell, 2011; Russell & Bohan, 2016).

Men's involvement in VAW prevention and feminist activism has consequently been a longstanding site of political contestation, especially in the early women's feminist movement (Harding, 1987; Hester, 1984). The early focus on women as the primary agents for change concerning gender injustices and men's violence originated from concerns that the inclusion of men—as actors potentially invested in maintaining the dominant gender order—could jeopardize the core aims of the movement, particularly at its inception. The gender identity of those engaged in the movement consequently became heavily imbricated with the social justice objectives of the movement itself (Lehrner & Allen, 2008). The concerns expressed turned on the threat posed to the security of gendered political and epistemological territories, manifesting in the potential for the co-optation of the movement, the obfuscation of women's voices and the curtailment of their decision-making capacities. All are connected to questions regarding how men negotiate their presence within women-only or women-majority (movement) spaces (Case et al., 2012; Lewis et al., 2015). These concerns persist in debates about men's participation in the women's VAW movement, incorporating the potential for depoliticization of the movement (Burrell & Flood, 2019) and undermining its transformative agenda (COFEM, 2017; Flood, 2011). A failure to acknowledge the role and function of gender inequalities in men's VAW is another ongoing concern, and tensions regarding the allocation of already limited resources feature heavily (COFEM, 2018). The risk of men's “pedestalling” (Macomber, 2015), where men are disproportionately praised for their contributions to anti-VAW practice or activism in particular, represents another challenge.

Approaches to engage men and boys in anti-VAW activism often incorporate the strategic instrumentalization of men's relatively privileged status to effect social change (Macomber, 2014). For some, this functions as a mechanism to overcome the barriers associated with men feeling “attacked” or “guilt-laden” by feminist messaging or those that confront notions of unearned privilege (Messner, 2016). But while this approach has, in some circumstances, successfully engaged men, it can also risk replicating or reinforcing masculine privilege in efforts to address men's violence (Casey, 2010). The maintenance of systems of privilege within these types of settings is indicative of what has been termed “hierarchical drift,” enabling the reinstatement of a dominant-subordinate status binary, as Russell (2011, p. 390) discusses in relation to heterosexual peoples’ allyship with LGBTQ + people. Crucially, it can produce the decentralization of the disadvantaged group within the context of collective activism (Russell, 2011), which serves to re-entrench unequal statuses, based in this case on sexuality and gender.

Black Feminist scholarship provides some of the earliest and most incisive examples of coalition building across boundary lines of difference, both within the context of activism to address VAW, as well as racial and gender-based oppression (Collins, 1986; Lorde, 1984). While accounts of coalition building point to the difficulties associated with mobilization across differently positioned groups, they also elaborate on how unequal hierarchies can be productively negotiated within movement spaces. The value vested in recognizing difference rather than its elision is a central tenet of effective coalition (Johnson Reagon, 1983; Smith, 1983). Emphasis is placed on developing alliances across race, class, gender, and other markers of difference, grounded in mutual respect and empathy (Collins, 2012). In the context of engaging men, a meaningful coalition includes rejecting a framing of men's power and privilege as monolithic (Collins, 2000). Understanding gender identities as coalitional (Carastathis, 2013) can further broaden the potential for collaboration across gender differences, despite historical tensions, to work toward shared goals and in ways that are attentive to structural inequalities. Coalition, as Lugones (2003, p. 11) asserts, “rearranges both our possibilities and the conditions of those possibilities” as it transforms how individuals and groups perceive their capacity for change. Crucially, it has the potential to disrupt extant power structures and, in turn, reveal avenues for collaborative action that can enable new forms of resistance and solidarity.

## Methods

### Recruitment and Participants

Using purposive sampling, 57 people were recruited into three distinct participant groups: (1) women with lived experience of DVA (victim–survivors; *n* = 25), (2) women DVA practitioners (*n* = 18), and (3) “engaged men” (*n* = 14). The author used a participatory informed approach and an intersectional feminist theoretical framework. This paper focuses on the combined contributions of the two women's groups (*n* = 43). Findings concerning how the men participants in this study conceptualize their roles are discussed in a separate article (Wild, 2023). All participants were recruited via a project website, “gatekeeper” DVA organizations, and the author's involvement as a volunteer in two community projects, including a breakfast club for women involved in the British carceral system and a therapeutic craft group for women who had experienced DVA.

Participants took part in an unstructured, narrative interview and/or focus group. Located in seven towns and cities in England, they were aged between 22 and 74. There was limited diversity in the sample, with most describing themselves as White British, cisgender, and heterosexual. Three women of Asian and African heritages participated, along with two lesbians and one gender-fluid woman. Approximately two-thirds of the women with lived experience of DVA reported co-occurring support needs, including mental health challenges, financial insecurity, homelessness, involvement in sex work, and substance use. The limited degree of ethnic diversity in the sample was a significant shortcoming. The lack of representation of trans victim–survivors or practitioners was another significant limitation.

### Ethics and Risk Considerations

While there were distinct ethical considerations for each participant group, the ethical imperatives bound up with this study were most pronounced in the case of participants with lived experience of DVA, owing to the potential risk of harm resulting from taking part. The study was trauma-informed and conducted with an ethics of care and reciprocity that prioritized harm minimization, well-being, and nonextractive ways of working (Campbell et al., 2019; Oakley, 2016). The author recognized the situated nature of the study via a continual process of self-reflexivity, which included acknowledging the extent to which her own intersectional identity, biases, and lived experience of childhood DVA inevitably shaped the research. The author was alert to the importance of addressing the relational power dynamics in research, and as such, participants were understood as collaborators and experts in their own lives, enabling the co-construction of the knowledge produced (England, 1994). Building on this perspective, the approach to risk and ethics management was rooted in “positive empowerment” (Downes et al., 2014). Participants were therefore located as active stakeholders at all stages of the research, with emphasis placed on their agency, control, and decision-making capacities, especially those participants with lived experiences of DVA.

The ethics process was understood as emergent, iterative, and dynamic in nature, with consent procedures being revisited at different points during the study. Participants were supported in making informed decisions regarding if, how, and when they took part in ways that felt appropriate for them. Well-being measures included providing information for free and confidential support, “signposting” and assistance with onward referral to specialist services, access to a dedicated project phone number and email address, and post-interview “check-ins” at different points in time. An assessment of the participant's safety and well-being was conducted before the interview; this included ensuring they were in a place of safety, at no risk of harm, and carefully establishing if participation could lead to distress. In a few cases, people who contacted the study in crisis were not invited to participate; instead, they were offered “signposting” and information about how to engage with the study in alternative ways.

A key aspect of the research design was the inclusion of a Project Advisory Group (PAG), a self-selected group of women participants with lived experience of DVA who acted as project advisors. The incorporation of a PAG is consistent with an inclusive, participatory-action feminist methodology (Reid, 2004), which emphasizes the removal of barriers to participation and inclusion while also increasing researcher accountability (Liddiard, 2013). All participant names have been altered to protect their anonymity, and uniquely identifiable information has been omitted. Details such as location, role, or organization of practitioner participants are deliberately broad or absent to maintain confidentiality and avoid detection (Sullivan & Cain, 2004).

### Discourse Analysis

The approach to discourse analysis adopted in this study was informed by an analytic tradition that understands talk and text as interactional, action orientated, and constructive of the social worlds we inhabit (Potter & Wetherell, 1987; Wood & Kroger, 2000). The focus was on what is accomplished by the participants’ narrative as a key discursive resource mobilized in their sense-making of “engaged men” or men's role(s), based on lived experiences. Data were transcribed using an amended version of the Jefferson (1984) convention, including recording silences and irregular speech. “Cleaning” of the transcripts (Elliott, 2005) was avoided as much as possible to increase the participants’ visibility in the discourses they mobilized. Data were coded and analyzed by identifying discursive patterns present in the data in the form of account variability and consistency. Variability concerns the differences in form and content of the accounts analyzed, while consistency is related to the variety of discursive features shared in and across the accounts (Potter & Wetherell, 1987). Attention now turns to a discussion of the findings from the discourse analysis of participant accounts.

## Findings

This section explores the five primary themes that emerged in the data and the ways in which they were discursively constructed in participant accounts, drawing on learning from the discourse analysis. These include: (i) the imperative of men speaking out against men's violence; (ii) the presence and function of “men listen to men” discourses; (iii) intersectional (re)framings concerning which men are engaged as anti-VAW actors; (iv) the risk of “pedestalling” and co-optation; and finally, (v) the potential for repair and restoration associated with men's participation. This paper will now progress to the first of these themes.

### “Far Too Much Onus Is Put on Women”: The Imperative of Men Speaking Out

Accounts discussed in this paper unanimously supported the notion that men do have a part to play in preventing men's violence, first and foremost by using their voices and positionalities to speak out and publicly denounce it. This assertion is based on a recognition that men must confront a culture of men's silence, and one in which women continue to be held disproportionately accountable for raising awareness about men's VAW—especially S/DVA—as well as for addressing it, with far too little attention placed on men and society more broadly:

Well, [men] do [have a role], don't they, because it's them that's doing it. […] I mean they need to […] speak out, don't they? (Aileen, age 57, White, heterosexual, lived experience group [LEG])

The message [for] me, should be (..) that the onus…far too much onus is put on women, through this experience, this journey, and the perpetrator […] the perpetrator seems to, not learn anything. (Marion, age 58, White, heterosexual, LEG)

These women's accounts reflect a core logic for men's engagement, offering a common sense rationale grounded in the reality that men are the majority perpetrators of VAW, and women disproportionately victim–survivors. Here, the advantage of men “speak[ing] out” lies in the potential for shifting responsibility away from women, minoritized genders, and victim–survivors. Another is that men's engagement could catalyze the behavior change among their peers, including those who abuse.

A similar argument was discernible in one of the focus groups with women who had experienced DVA (*n* = 8), enrolled on a DVA support program:

Hannah: I think speaking out is kinda important, really.

Hannah: Making men more aware.

All: ((in agreement))

Author: And why do you think that it is?

Kirsten: Still goes back to years ago doesn't it … male domination—

Hannah: It's their behavior that needs to change […] [if men were] more aware […], then a lot of these [things] that are happening to victims, wouldn't happen. (ages 25–39, LEG):

When describing men's collective role, the women mobilize a discourse of men's violence as a gendered social problem rooted in historically embedded oppressions of women. Also present is the recourse that non-violent men are not “aware” of the problem. One of the substantive gains of men actively challenging VAW is to improve outcomes for victim–survivors and to address the structural factors that create the conditions for men's violence. This includes disrupting a perceived general lack of awareness about the problem among men. In offering these arguments, the women allude to the need for men to participate meaningfully in activities aimed at disrupting broader patriarchal relations as part of the ongoing work to challenge men's violence(s). Often, discourses that incorporate the notion that men listen more readily to other men are mobilized to achieve men's entry into this work, as the next section outlines.

### “What Message Are Men Giving to Men?”: “Men Listen to Men” Discourses

The imperative for men to speak out frequently intersects with discourses that position men as uniquely placed to influence or reach their male peers. These “men listen to men” discourses were present in several accounts, reflecting assertions made by the “engaged men” who took part in this study (Wild, 2023), as well as elsewhere (Casey et al., 2017; Messner et al., 2015). Their recurrent importation speaks to the strategic mobilization of men's epistemic authority, broadly scaffolded by a gender-inequitable society that routinely treats the voices of women and gender-minoritized people as secondary to those of cisgender men. In practice, “men listen to men” discourses can accomplish divergent and opposing outcomes—both positive and negative—concerning men and boys’ engagement, as analysis here shows. Crucially, they can function to relocate the “onus” (Marion) for modelling nonviolence and/or for challenging violence-supportive norms and attitudes with men, as one participant states: “What message are men giving to men? The non-violent men giving to the violent men. They’re not giving any really, are they?” (Dawn, age 37, White, heterosexual, LEG)

The argument follows that it is incumbent on “nonviolent” men to harness their influence to educate themselves and other men—including those that harm—about the unacceptability of VAW or GBV more broadly, thereby fostering a type of relational accountability within the context of a (hetero)masculinized culture. The (re)allocation of responsibility this entails is a significant advantage of a “men listen to men” discourse, as it functions to redistribute some of the labor of contesting men's violent or misogynistic talk and practices onto men. It also creates vital opportunities to disrupt the performance of certain (hetero)masculinities that are harmful and which tacitly perpetuate a culture of men's silence:

[Men] need to talk about [men's violence] […]. But they don't do they. […] You know, when I'm discussing other couples' [relationships] with my partner – he's a typical “Alpha male” – […], he's like “we don't discuss that; that's off limits (.) That's none of my business.” (Felicity, age 43, White, heterosexual, practitioner group [PG])

Felicity argues here that it is men who must challenge framings of VAW as a “taboo” subject among men or one that does not apply to them. Imported in the above quote is a historicized discourse that relies on the construction of VAW as women's prerogative, legitimized and made coherent via an appeal to a (partially) outmoded framing of certain types of GBV, such as DVA. DVA is constructed as an individualized, discrete, heterosexual phenomenon between a woman and a man, isolated to the domestic sphere. Felicity's account is simultaneously orientated toward a discourse of “hegemonic masculinities” (Connell & Messerschmidt, 2005) as she invokes the figure of the “Alpha male.” This is coupled with an implicit reference to men's perceived inability to “talk about [things],” a proclivity characteristic of emotionality and therefore relegated to women and feminized bodies.

“Men listen to men” discourses turn on the notion that men have unique access to other men and men's spaces based on a shared gender identity. For some participants, this enables “off limits” (Felicity) conversations to be pursued in a manner otherwise impossible if initiated by women, as practitioner Mudiwa describes:

I think men can be followers. I think sometimes if they have another man they respect talking about stuff, they sometimes pay attention to it. If you had a woman preaching at you, telling you about GBV, you'd be like, what? What are you on about woman? (Mudiwa, age 37, Black, heterosexual, PG)

According to this formulation, the gender politics and unequal power relations often underpinning the perpetration of men's VAW are deemphasized in favor of leveraging men's social networks and peer relationships as a strategy for their engagement. This framing gains legitimacy owing to the perceived derogatory masculine lens through which women are constructed as “preaching” subjects. In advancing this discourse, Mudiwa arguably makes a “patriarchal bargain” (Kandiyoti, 1988) by acknowledging the strategic gains of capitalizing upon men's epistemic authority and the mutual respect men are understood to afford (some) men.

While these participants espouse the merits of “men listen to men” discourses, data also pointed to the ways in which they can produce less favorable outcomes, chiefly because they sustain a narrow epistemological frame that is patriarchal and which centers the primacy of men's voices (Alcoff, 1991). Consequently, it is imbued with the potential to replicate, preserve, and/or deepen the very gender inequalities men's engagement seeks to disrupt:

That's the problem [for] me…If you're a man that can't do that […]; that can't listen to a woman and listen to her authority. Her real experiences and believe and trust and have an emotive response to that. If it takes a man to hear it from another man, for him to be engaged, then he shouldn't even be engaging. (Amber, age 22, White, heterosexual, PG)

Amber's account taps into a broader discourse, which denotes the dangers of reproducing a cultural narrative that is inherently dismissive of women's voices simply because they are women. The attendant gender politics are problematized further via a formulation organized by and reliant upon sustained structural inequalities and binarized gender. It also provokes critical interrogation of not only which men ought to be doing the speaking but also to whom they should speak, as elaborated in the next section.

### “Men Who Stand Up Tend to be ‘Hippy Do-Gooder Types’”: Intersectional (Re)framings

The question of which men should or could be engaged surfaces in different ways in the data and is modulated by diverse social and political identities that converge to create different modes of privilege, power, and access between and among men and masculinized bodies. This reflects the stratification evidenced in prevention and intervention approaches within which the “engaged man” is envisaged and constructed; typically homogeneously White, cisgender, and heterosexual (Flood, 2015). Abigail alludes to this construction as she discusses the hypothetical engagement of her abusive ex-husband:

We don't have enough men standing up. And I think also because of the way society is, the men who do stand up, they tend to be seen as a bit, like “hippy do-gooder types.” And we could do with more rugby player, football, butch, muscly men, you know. Who work out in the gym […], just to be in the front going, “no that's the wrong way. This is how you are a man” […]. And I think it's important for men to see that in other men. Because if you've got [to engage] a bloke like my ex; he's a typical football-loving, beer drinking bloke […]. (Abigail, age 51, White, lesbian, LEG)

Abigail's account underscores the assertion made by some masculinities scholars that men's own social locations strongly inform their capacity to engage with prevention work (Flood, 2015). Akin to earlier accounts, Abigail's talk is squarely located within a heteromasculinities framework of analysis in which “real men” tropes provide the requisite resource capital for catalyzing the enlistment of otherwise disinterested men—including those who use violence themselves. Set within the broader discourses of “men listen to men” referred to earlier, the men who perform these masculinities are constructed both as the rightful “target” for violence prevention programming, as well as the cohort of men who should be more involved in anti-VAW organizing, if broader sociocultural change, as well as better outcomes for victim–survivors, are to be achieved. It is an identity performatively achieved through the enactment of patriarchal heteronorms and is constructed in Abigail's account as antithetical to the “hippy do-gooder types” believed to be involved in the anti-VAW movement. While shining a light on the strength of these masculinities, Abigail advances a latent discourse concerning the social and political identities of the men concerned: politically left-leaning, White, heterosexual, middle class, and thus incapable of reaching men such as her ex-partner.

By way of contrast, practitioners spoke about the need to disrupt patriarchal heteronorms rather than instrumentalizing them in an attempt to engage more men:

We should not gender boys to be strong, not in touch with their emotions; when they're angry [say]; “oh, boys will be boys!” […] I think men play into it…Can't do washing, ‘cause don't know how washers work, and yet they're all fine engineers, and it were men that put men on moon…But they can't work a washer? (.) Culturally, we let them off, don't we? (Debbie, age 54, White, heterosexual, PG)

A familiar discourse of heteronormative gender socialization is imported again by Debbie to refer to the routinized, everyday practices which enculturate men and boys as emotionally disengaged subjects, incapable of care or domestic work. It also reflects the assertions made by the men participants engaged in this study (Wild, 2023). While this discourse arguably no longer has the strength it may have had in earlier decades, its mobilization exposes two key contentions. First, the often woefully low societal expectations placed on men and boys to behave in ways that challenge these norms—culturally, should we not expect more of men's ability to act beyond these limiting frames associated with their gender role? Second, it is a discourse incompatible with other widely circulating discourses regarding men's intellectual or logistical capabilities when operating outside of the domestic sphere. Certain behaviors are instead rationalized as the product of an immutable “nature” embedded in dominant gender identity constructions, thereby foreclosing critical scrutiny and restricting possibilities for structural change, especially regarding the root causes of VAW and GBV more broadly. These pitfalls coalesce with the risk of men's “pedestalling,” leading to possible co-optation as discussed in the section that follows.

### “It’s Just Really Tokenistic”: “Pedestalling” and Co-optation

Men's engagement and participation in the anti-VAW space were not unambiguously promoted among participants, even if all the women agreed there was a role for men. Some expressed uncertainty regarding men's motivation for becoming involved, especially when occupying roles in public-facing campaigns. In accounting for their concerns, some participants mobilized a discourse of tokenism which, as data here show, closely connects with the risk of men's potential “pedestalling”:

[There were] loads of male heads within the council posed with this [cardboard cut-out], and it's just really tokenistic, like, it doesn't…what's that really going to achieve? The money that they spent on that campaign and spending all that time going around the heads of the council could have been much better spent doing a few workshops in a school. Just sometimes feels a little like, “oh we've ticked that box now, the public can see that we're against domestic violence, now let's crack on with something more important.” (Erin, age 36, White, heterosexual, PG)

I can't see [men] doing anything else other than […] the bit that makes them look really good, “up there” in their suits. (Angela, age 43, White, heterosexual, PG)

These practitioners are critical of gesture politics based on what they view as a purely symbolic, perfunctory participation in the movement to resource a public image as “good” men. In so doing, the men concerned are regarded as fulfilling bureaucratic responsibilities without accomplishing meaningful political or social change, thereby subtly depoliticizing the work (Lehrner & Allen, 2008). Erin's account also speaks to the political deprioritization of the interests of women and minoritized genders, in favor of “something more important,” thereby reinforcing charges of tokenism. With this is a concealed reference to the already hostile funding environment VAW services must navigate, a situation greatly exacerbated by the emergence of COVID-19. Implicit in this are questions regarding how expanding men's engagement will be adequately resourced.

A minority of practitioners expressed suspicion regarding men's primary motivation for participating in the antiviolence movement. Concerns regarding the potential for men's spurious or malicious involvement seemed to be more keenly felt among women with a longer history of practice in the sector, such as Angela, who stated, “Some [men] might be in it for the wrong reasons […] I think you have to be very, very cautious.” Coinciding with this were anxieties regarding co-optation:

I think there is a fear, you know, with women, when men do start to come in, because…especially when you're fighting for something that is so gendered. You sometimes might find it a bit difficult to trust these blokes that are involved, you know. We all know that there are men that pretend to be these…(.) “right on” socialists, when actually it turns […] they're using it as a disguise. […] And there's a power behind a sisterhood, isn't there? (Amber, age 22, White, heterosexual, PG)

In evidence here, is a comparable discourse to the one expressed by Angela and Erin. It is reminiscent of warnings expressed by feminist activists during the nascent stages of the movement, regarding the potential for men's engagement to decenter women's experiences and interests, as well as risking further fragmentation of an already fragile space (COFEM, 2018; Lehrner & Allen, 2008). At the same time, a discourse of solidarity based upon a shared gender identity is discernible here, not only as a source of epistemic authority but also of (physical and political) security. Jean mobilized a similar discourse, orientated toward the value vested in a shared gender experience:

A woman wants to talk to another woman. Not a man. Do you know what I mean? They'll feel more easy with it being a woman…'specially some of them men, what…the abuse that men have gone to. […] Could think um…the man that you're speaking to is abusing his wife at home. […] – people are good at putting up a front. (Jean, age 45, White, LEG)

Jean's account, like Amber's, is reminiscent of a particular feminist logic, which argues for a unique understanding between and among women based upon a shared experience of oppression. For Jean, collective experience is bifurcated along gender lines; gender sameness cultivates possibilities for relatability, mutual understanding, and crucially, a felt sense of safety after experiencing men's abuse. A preoccupation with men's “true” intentions is also salient in this account, which, for Jean, originates from a possible dissonance between behaviors in private and those in the workplace, thereby reflecting uncertainties expressed by practitioners Angela and Amber before her. Critically, the significance of trauma in shaping some women's thinking concerning “engaged men” is also notable here, as set out in the next section.

### “If You Got Talking With a Man, You'd Just See His Fist”: The Role of Trauma

For some participants with lived experience of DVA, discourses of uncertainty and skepticism critically intersect with one of fear and trauma when considering men's role. The strength of this discourse is further embedded in the context of having lived through DVA, an experience that can fundamentally undermine one's sense of personal safety and self-identity (Herman, 1992), as this account illustrates:

I can't really go on a train, me. I can't go on a bus, I'm scared. It's men. Yeah, scared of men. Yeah, I get anxious. […]. I don't want… no, no, no… because you can't trust again […]. If even you got talking with a man, you'd just see their fist, or whatever, do you know what I mean? […] That's all you're going to expect. (Aileen, age 57, White, heterosexual, LEG)

It is starkly evident here that Aileen's fear is in no small part constituted by her experiences of DVA, which have significantly impacted her ability to engage with or relate to men. The account of her daily existence exemplifies a series of complex trauma reactions to the extent that seeing a man functions as a catalyst for the “reawakening” of her trauma (Herman, 1992). The depth of her distress is reflected in her fragmented account, which for Aileen forecloses the possibility of “trust[ing]” a man, including in a professional capacity. It must be noted, however, that while Aileen felt unable to engage directly with men, she explicitly stated elsewhere in the interview that men do have a role, thus underscoring the imperative for nuance when considering the question of men's participation and the form it takes. Substantiating this claim were accounts that spoke to the potential benefits associated with men's participation, especially at the level of the individual, as elaborated in the section that follows.

### “He Restores Your Faith in Men”: Repair and Restoration

The gains associated with men's participation in victim–survivor facing practitioner roles are also evident in the data, as some participants import a discourse of repair and restoration. Men's involvement in the field is instead construed as a tool for some victim–survivors’ recovery:

I think he restores […] your faith in men. You realize that not every man is an asshole basically. And that there are genuine men out there. Because he's quite laid back as well, you do feel that you can talk about anything with him. (Kirsten, age 27, White, heterosexual, LEG)

I think [men working in the sector is really positive. I wish there were more male workers. I understand why women would prefer to see a female worker initially, but I think in terms of breaking that cycle, they also need to see what a… have positive male role models. And it's not thinking that all men are bastards, you know, there are actually nice ones. (Gemma, age 35, White, heterosexual, PG)

The first extract is from a focus group conducted with one of the few women's DVA support groups in England cofacilitated by a woman and a man, both of whom participated in this study. The account illustrates how the male facilitator's engagement with the group functions as a resource to destabilize negative constructions of men as adversarial or a homogenous group of potential perpetrators. Some practitioners echo this framing, as the second extract from Gemma indicates, as she imports the figure of the “positive male role model.” It is a trope in evidence across the sample, typically used to support a belief that men should take steps to rework their relationship to dominant gender roles and expectations that, in this case, incorporate professional roles that challenge or address men's VAW.

Further corroborating this point, analysis of the benefits of men occupying such roles indicates how they are closely enmeshed with the facilitator's own negotiation of and relationship to patriarchy, his perceived distancing from certain forms of masculinities, and his corresponding gender presentation. Together, these become key factors in the women's ability to engage with him as Hannah, another member of the same focus group states: “You don't even notice the fact that he's a man […] He's just one of us. It's when he leaves, he's a man again, in here, he's just one of us.” Here, Hannah offers a variant of the discourse deployed by Kirsten as she speaks to a type of identity work, which permits a temporary equivalence between the women and the facilitator within the boundaries of the closed group space. Functioning to create a climate of trust and safety, this gender identity suspension enables the women to position the facilitator as “just one of us.” Notably, the facilitator had lived experience of childhood DVA that he disclosed to the group (see Wild, 2023) that may have bolstered their confidence and sense of trust in him. With these findings in mind, this paper now progresses to a discussion of what they could mean for the theorization and conceptualization of men's role(s) in the prevention and intervention of men's VAW and GBV more broadly.

## Discussion

Participant accounts discussed here speak of the ubiquity of men's violence against the backdrop of a society that still struggles to get to grips with GBV and in which epidemic levels of men's violence continue to be tolerated. This reality provides a compelling justification for men's increased and proactive involvement in efforts to address GBV. Implicitly discernible in the formulations offered by the participants is the notion that men's inaction, regardless of their own individual behaviors or belief systems, equates to complicity. It is, therefore, imperative that those who do not abuse or use violence act. However, echoing studies concerning antiracist mobilization (Droogendyk et al., 2016), the women emphasize that they do not want to hold (primary) responsibility for educating or reforming men's harmful or violence-supportive beliefs, the shifting of wider societal opinion, nor for catalyzing men into action. Instead, they argue that men must shoulder some of this burden, including by taking on aspects of the emotional labor associated with this complex and often relentless work.

### Negotiating Epistemic Hierarchies to Achieve Change

As data have indicated, some of the most prominent discourses imported by participants to resource the imperative of men's increased responsibilization are “men listen to men” discourses, reflecting a commonly held belief that men are more motivated to take a stand against VAW when encouraged to do so by other men (Levtov et al., 2014; Peacock & Barker, 2014). The participant accounts point to how these discourses fulfil multiple functions, including countering victim-blaming or “authentic” victimhood narratives, which can leave those with lived experience of GBV feeling culpable and further stigmatized. Data also point to how the deployment of these discourses can function to secure men's pathway into violence prevention work or interest in gender-based issues, thereby echoing findings elsewhere (Bolton et al., 2024; Casey, 2010). In this sense, men's peer group interaction can function as an “enabling site” within which issues concerning gender and GBV can be critically and productively explored (Chopra, 2006).

However, this argument also represents one of the primary pitfalls associated with “men listen to men” discourses. This is because they rely on maintaining dominant gendered epistemic hierarchies, nurturing knowledge frameworks rooted in patriarchy, and turn on the construction of men as “elite knowers” (Janes, 2016). Their success points to a broader societal reluctance to accept women's epistemic authority, including regarding her own lived experience, and in this case, could risk slipping into a type of “allyship” in which people with privilege do not learn to listen, nor listen to learn (Carlson et al., 2020). Data indicate that participants, especially practitioners, register these concerns, as “men listen to men” discourses are not uncritically mobilized. Instead, their accounts expose the tensions concerning the extent to which “hegemonic” (hetero)masculinities should or should not be leveraged as a mechanism for men's engagement and change-making among men (Jewkes et al., 2015). As Gibbs et al. (2015) assert, if the mobilization of these masculinities reifies potentially harmful tropes, in practice, it could contravene or obstruct any gender equality aims associated with the engagement work undertaken.

This taps into a broader question concerning which masculinities should be instrumentalized, if at all, and by whom. A question made evident in the accounts of lived experience participants when discussing their own abusive partner's hypothetical or imagined engagement in efforts to prevent men's violence. They argue instead for a more expansive understanding of dominant masculinities and the identity factors used to resource them that cut across class, economic, and racialized boundary lines of difference. Their assertions strongly resonate with those of the men practitioners and activists engaged in this study (Wild, 2023) and serve as a reminder that men's awareness-raising or engagement initiatives often struggle to attend to differences among men, instead erroneously treating them as a homogenous group. This severely limits the scope and extent to which a more diverse range of men might be brought into GBV prevention, as participants allude to, while also underscoring the value and merits of an intersectional approach when strategizing men's engagement, as others have argued (Boonzaier et al., 2020; Peretz, 2017, 2018b).

For some practitioners, concerns associated with “men listen to men” discourses rest with the threat posed to women's leadership and stewardship of the VAW sector, echoing findings elsewhere that women's roles “could be supplanted or diminished by men's participation” (Tolman et al., 2016, p. 3). As such, a discourse that prioritizes men's willingness to listen to other men can silence the voices of women and gender minorities in practice spaces and potentially discount them as credible or authoritative voices on how to address VAW and GBV more broadly (Epstein & Goodman, 2019). This risks undermining the work of women and gender minorities in the sector (Carlson et al., 2020) if not conducted in a manner that is highly attuned to the extant gender power dynamics in operation. A key tension in violence prevention work with and by men is thus exposed: Should men be met where they are in order to “get them in the room” of violence prevention work (Casey et al., 2017) or should the work endeavor to transform extant gender norms and relations via the engagement work undertaken (Flood, 2015).

By engaging in activities that fundamentally challenge certain forms of masculinities and the dominant (gender) order, men arguably contribute to dismantling the system from which they routinely benefit, albeit not all men in equal measure (Peretz, 2017). This notion led some participants to question men's motivations for participating in this space in the first instance, particularly when conducted on a public stage or in public-facing roles. They argue that this type of participation can produce men's disproportionate valorization or “pedestalling” (Macomber, 2015), especially when it is not accompanied by a longer-term strategy or the resources necessary to achieve meaningful structural change. Salient here is how symbolic public gesturing by men as “allies” can hinder the movement's expansion and present obstacles to real change (Peretz, 2018a; Westmarland et al., 2021). Tensions are exacerbated further when considering the extant insecure and volatile funding environment for GBV prevention and intervention, and the potential diversion of funds away from women's or women-led organizations (COFEM, 2018). Together, the participants’ accounts, therefore, illustrate the complex and oppositional engagements with “men listen to men” discourses.

### The Importance of Listening to Victim–Survivors

Data strongly substantiated the importance of understanding and attending to the complex gender relations in operation in movement and practice spaces, as well as beyond them, and of earnestly listening to women and minoritized genders with lived experience of men's GBV(s). A limited number of women in the sample, all of whom had significant histories of abuse by men, painfully emphasize the risks of (re)traumatization attached to direct engagement with men practitioners, for some victim–survivors. Their accounts were fundamentally shaped by trauma, which contributed to a legitimate sense of uncertainty, fear, and distrust. These women's accounts, such as those of Aileen and Jean, should not be marginalized in the discussions regarding the sector's diversification to include men. Instead, their experiences must be centered, and the impact of trauma explicitly acknowledged. However, while these participants described how they felt unable to work with men on an individual basis, they maintained a strong belief that men do have a role to play in achieving gender equality, challenging harmful gender norms, and contributing to systemic change to eliminate VAW and GBV more broadly.

There is also important learning to be taken from the contributions of those participants who did endorse working with men in practitioner roles based on their experiences of receiving support, as discussed during one of the focus groups. While the discussion initially implied an uncomplicated acceptance of men's participation within the group space, upon closer analysis, it becomes clear how the women, despite fully endorsing the specific man they worked with, were cognizant of the contentions his gender identity status brought with it. His nonjudgmental, uncompromising recognition of the harm caused to them was read as a display of allegiance (Butler, 2004), which in turn cultivated a space in which the women's experience of men's violence is not only heard but also affirmed (Herman, 1992).

Taken together, participants’ accounts reveal there is a subtle yet important distinction when mapping out the types of roles men occupy in antiviolence work or activism. They are varied and can be drawn along the lines of individual interventions in practitioner or victim–survivor-facing roles versus collective, awareness-raising or activism roles at broader scales. Contributions from victim–survivors who have received direct support from men in practitioner roles indicate the potential advantages of men's participation in violence intervention work while noting the caveats concerning trauma discussed earlier. Data also indicate that the women's willingness to engage with men in these types of roles at times resides with the men's rejection of, or individual relationship to, patriarchy and “hegemonic” (hetero)masculinities. A crucial aspect of men's role in addressing men's violence(s), therefore, seems to incorporate challenging dominant constructions of masculinities and working to forge respectful, equitable, collaborative links with women in the movement based on a shared aspiration to create alternative ideas about what it means to exist today as a gendered being.

## Conclusion

The analysis reveals that despite the epistemological, practical, and political challenges that can accompany men's participation in efforts to address men's VAW and GBV more broadly, there is clear consensus regarding the urgent imperative for more men to take a proactive stance in ending men's violence(s). Participant accounts strongly corroborate that men should occupy a more visible role alongside women and minoritized genders in broad-ranging efforts to address GBV, including by raising awareness among other men and boys, and through talking to their peers. Men must also support the rights of women and minoritized genders to speak freely about these issues without fear of retribution or misogyny. Bound up with this is the need to recognize that GBV cannot be eliminated without fundamentally reworking socially enculturated, patriarchal discourses concerning men's violence(s) and (hetero)masculinities that, if left unchecked, can minimize, normalize and/or perpetuate gendered violence.

“Men listen to men” discourses are well-rehearsed and can offer a pragmatic pathway to initiate men's involvement while responding to calls for men to speak out and disrupt narratives of victim-blame. However, the challenges of these discourses are evident when operationalized in practice. While they capitalize on men's influence in a patriarchal system to catalyze greater participation, they can also become a mechanism to preserve men's elevated position within an epistemological hierarchy that demotes the contributions of women and minoritized genders, sidelines their decision-making capacities and/or silences their voices. Thus, the data underscore the importance of men undertaking anti-VAW work that is alert to historical gender oppression and that fosters women's ongoing leadership in the GBV sector so that men's involvement does not replicate extant patterns of privilege. Coalition with women underpinned by adequate, sustainable resourcing and long-term strategic planning, coupled with a commitment to structural change led by women and minoritised genders, is critical to prevent tokenistic involvement and ensure that men's contributions are substantive rather than symbolic.

Findings also emphasize the necessity of an intersectional and diversified approach to men's engagement so that a much wider range of men can take up a role in addressing men's VAW and GBV more broadly. Strategies that rely only upon the instrumentalization of hegemonic masculinities as vehicles for engagement and change making among men are particularly fraught, as they risk reifying norms that could undermine gender equality. However, it cannot be overlooked that some participants strongly felt that these norms might be one of the few ways of reaching certain men who exist on the peripheries of conversations about gender or GBV and/or who experience some form of marginalization or minoritization themselves. Crucially, however, this approach should be understood as just one within a range of measures to address GBV and other gender-based issues and inequalities, including how men and boys experience gender socialization.

Finally, excessively narrow framings of men's roles as “allies” cannot fully accommodate the realities of trauma and how a lived experience of men's violence and abuse fundamentally influences the potential for alliance. This exposes the limitations associated with an “allyship” model shaped by those with structural advantage. To counter this, this study suggests there is a need for coalition that fully addresses intersectional and gendered power differentials and which is underpinned by an empathic, nuanced understanding of trauma and the interaction with patriarchal violence. Crucially, the coalitional anti-VAW work undertaken must incorporate accountability at various scales. First and foremost, this is achieved by listening to victim–survivors, especially when considering practice-based roles, and consistently centering their lived experience epistemologies in the design of policy and practice interventions.
